# Extracellular Vesicles Associated Metabolites as Intercellular Signalling Mediators in Disease and Therapy

**DOI:** 10.3390/metabo16030207

**Published:** 2026-03-20

**Authors:** Abdul Qadeer, Abd Ullah, Muhammad Zahoor Khan, Khalaf F. Alsharif, Fuad M. Alzahrani, Khalid J. Alzahrani, Abdulwahab A. Abuderman

**Affiliations:** 1 School of Medical Sciences, Shandong Xiehe University, Jinan 250109, China; 2 College of Agriculture and Biology, Liaocheng University, Liaocheng 252000, China; 3 Department of Clinical Laboratories Sciences, College of Applied Medical Sciences, Taif University, P.O. Box 11099, Taif 21944, Saudi Arabia; alsharif@tu.edu.sa (K.F.A.); fuadmubarak@tu.edu.sa (F.M.A.); ak.jamaan@tu.edu.sa (K.J.A.); 4 Basic Medical Sciences Department, College of Medicine, Prince Sattam Bin Abdulaziz University, Alkharj 16273, Saudi Arabia; a.abuderman@psau.edu.sa

**Keywords:** extracellular vesicles, metabolome, metabolites, intercellular signaling, immunometabolism, tumor microenvironment, hypoxia, drug delivery

## Abstract

Extracellular vesicles (EVs), particularly exosomes, have emerged as critical mediators of intercellular communication, yet the metabolite fraction of their cargo remains substantially underexplored relative to proteins and nucleic acids. This review synthesizes current knowledge on the exosomal metabolome as a functionally distinct intercellular signaling system with unique biophysical properties. We review the mechanisms proposed to govern metabolite encapsulation into exosomes, encompassing membrane transporter involvement, lipid raft partitioning, and binding to luminal proteins, and discuss the unresolved question of whether metabolite loading is selective or stochastic. Critically, we present a quantitative framework evaluating whether delivered metabolite quantities are sufficient to alter recipient cell metabolic pools, distinguishing receptor-mediated signaling from bulk substrate delivery. We also address methodological considerations including contamination artifacts and isolation-method biases that complicate interpretation of EV metabolomics data. Exosomal metabolites are reviewed across four functional categories: energy substrates (ATP, lactate, amino acids), signaling molecules (TCA cycle intermediates, eicosanoids, nucleotides), redox cofactors and antioxidants (NADH, glutathione), and oncometabolites. For each category, available evidence is critically appraised, distinguishing metabolites with direct mass spectrometric detection from those whose roles are inferred from parent-cell biology. The review examines the roles of exosomal metabolites in tumor-stroma metabolic symbiosis, immunometabolic regulation, inter-organ crosstalk in metabolic diseases including type 2 diabetes and non-alcoholic fatty liver disease, cancer metastasis, viral infections, and immune evasion. A quantitative framework is discussed to evaluate whether delivered metabolite quantities are sufficient to alter recipient cell metabolic pools, distinguishing receptor-mediated signaling from bulk substrate delivery. Technical challenges in exosomal metabolomics are reviewed, including the impact of isolation method on data quality, contamination artifacts, and current standardization gaps. Therapeutic implications of exosomal metabolite signaling are discussed, encompassing metabolite-loaded exosomes as therapeutic vehicles and exosomal metabolite loading as a pharmacological target. Integration of single-vesicle technologies with systems biology approaches is highlighted as a promising direction for advancing this field toward precision medicine applications in oncological and metabolic disorders.

## 1. Introduction

Extracellular vesicles (EVs), particularly exosomes, have transformed our understanding of intercellular communication over the past two decades [1,2,3]. These membrane-bound nanoparticles (30–200 nm) are secreted by virtually all cell types and carry molecular cargo capable of modulating recipient cell function [4,5]. The landmark discovery by Valadi and colleagues that exosomes can transfer functional messenger RNAs and microRNAs between cells sparked an explosion of research focused primarily on their nucleic acid content [6]. Since Valadi and colleagues demonstrated that exosomes transfer functional mRNAs and microRNAs between cells [6], research has focused heavily on nucleic acid and protein content [7,8], inadvertently creating a blind spot: the exosomal metabolome.

**Figure 1:**
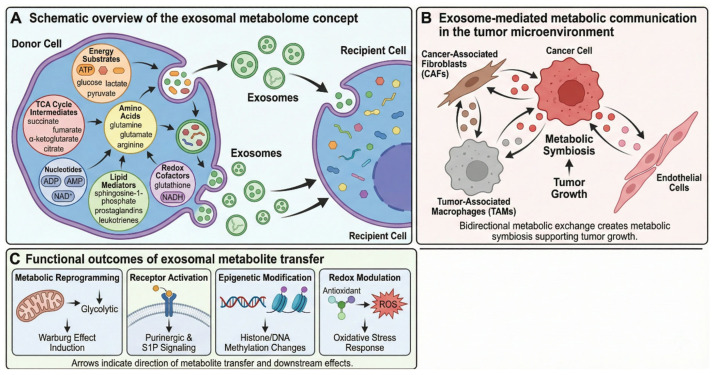
The exosomal metabolome: a new paradigm in intercellular signaling (**A**) Schematic overview of the exosomal metabolome concept. Donor cells package diverse metabolite classes into exosomes, including energy substrates (ATP, glucose, lactate, pyruvate), TCA cycle intermediates (succinate, fumarate, α-ketoglutarate, citrate), amino acids (glutamine, glutamate, arginine), nucleotides (ADP, AMP, NAD+), lipid mediators (sphingosine-1-phosphate, prostaglandins, leukotrienes), and redox cofactors (glutathione, NADH). Note: Metabolite classes are depicted schematically; direct exosomal detection by mass spectrometry has been confirmed for purine nucleotides [18], S1P [19], lactate and TCA intermediates [20], and eicosanoids [21], whereas redox cofactor representation is based on luminal enzyme activity studies [22]. (**B**) Exosome-mediated metabolic communication in the tumor microenvironment. Cancer cells release exosomes that reprogram metabolism in recipient stromal cells, including cancer-associated fibroblasts (CAFs), tumor-associated macrophages (TAMs), and endothelial cells. Bidirectional metabolic exchange creates metabolic symbiosis supporting tumor growth [20,23]. (**C**) Functional outcomes of exosomal metabolite transfer: metabolic reprogramming (Warburg effect induction), receptor activation (purinergic and S1P signaling), epigenetic modification (histone/DNA methylation changes), and redox modulation (oxidative stress response).

Metabolites represent the most ancient form of biological signaling, predating complex proteins and nucleic acid-based regulatory systems [7,8]. They constitute the primordial language of cells, and are rapid, dynamic, and pharmacologically potent [9]. Yet in exosome research, metabolites have been treated as passive byproducts. This review challenges that assumption, proposing that the exosomal metabolome functions as a primary signaling system complementing, and in some contexts superseding, nucleic acid- and protein-mediated communication (Figure 1). That said, direct comparative evidence for functional hierarchy among cargo classes remains limited, and metabolite primacy likely depends on context, metabolite class, and vesicle dose delivered.

Recent advances in mass spectrometry-based metabolomics and single-vesicle analytics have revealed the diversity and specificity of exosomal metabolite cargo, demonstrating that these small molecules are active signaling agents, not bystanders [10,11,12,13]. The field now recognizes that exosomal metabolites can function as bona fide signaling molecules, engaging specific receptors and modulating intracellular metabolic pathways in ways that are distinct from, and complementary to, protein and nucleic acid-mediated effects [14,15,16,17].

Four arguments support metabolite-based exosomal signaling as functionally significant. First, metabolites act immediately upon cellular uptake, requiring no transcription, translation, or post-translational processing, directly modulating enzymes, allosteric regulators, or cell surface receptors within seconds to minutes [24]. Second, they are pharmacologically active at physiological concentrations, modulating enzyme activities, serving as energy substrates, or activating GPCRs and nuclear receptors [25]. However, an important quantitative constraint must be acknowledged: whether metabolites delivered by EVs are present in quantities sufficient to meaningfully alter intracellular metabolite pools of recipient cells is not self-evident, and is discussed in detail in Section 2.3. Third, exosomal metabolite composition is highly sensitive to the parent cell’s metabolic state, enabling real-time transmission of metabolic information across tissues [20]. Fourth, from a mass perspective, lipids and small molecules constitute the majority of exosomal cargo yet receive disproportionately little research attention relative to trace RNA [26,27]. Lipidomic studies consistently show exosomes are enriched in cholesterol, sphingomyelin, and glycosphingolipids compared to parent cells [28]. Notably, metabolite, protein, and nucleic acid cargo most likely act in concert in most biological contexts, and the functional hierarchy among them remains an open question [10].

Clinical relevance spans multiple disease states. In oncology, exosomal metabolomics supports liquid biopsy approaches for cancer detection via cancer-derived metabolic signatures in accessible biofluids [29,30]. In high-altitude cerebral edema (HACE), serum exosomes show 26 significantly altered metabolites—including adenosine, guanosine, and purine pathway intermediates—reflecting systemic hypoxic adaptation [31]. In remote ischemic preconditioning, exosomal metabolites carry neuroprotective signatures [32]. In reproductive aging, follicular fluid exosomes show 17 significantly altered metabolites between young and older women, spanning amino acids, carbohydrates, fatty acids, and nucleotides [33]. In metabolic diseases, including T2DM and NAFLD, exosomal metabolites have emerged as inter-organ signaling mediators and early biomarkers of disease progression, as discussed in Section 4.6. Collectively, these examples establish exosomal metabolites as sensitive reporters of physiological state across health and disease.

This review comprehensively examines the exosomal metabolome as an intercellular signaling system, covering mechanisms of metabolite incorporation, functional metabolite classes, and roles in cancer, immunity, metabolic disease, and environmental stress adaptation. We address historical technical challenges and highlight recent breakthroughs in mass spectrometry and single-vesicle analysis [34,35], and discuss therapeutic implications including metabolically active vesicle-based therapeutics and novel pharmacological targets for modulating exosomal metabolite loading and release.

While prior reviews have addressed exosomal metabolomics compositionally and methodologically [10,36], the functional biology of metabolite-based exosomal signaling—spanning receptor activation, immunometabolism, epigenetic regulation, and redox biology—has not been integrated into a single conceptual framework. This review additionally incorporates recent advances in exosome biology in viral infection and metastasis; areas not previously covered in dedicated exosomal metabolomics reviews.

## 2. Biogenesis of the Metabolic Cargo: Mechanisms of Selective Incorporation

### 2.1. Pathways of Metabolite Encapsulation

Selective incorporation of small, often charged metabolites into the lumen of forming intraluminal vesicles (ILVs) involves multiple, partially overlapping mechanisms operating during exosome biogenesis within the endosomal system [1,4,37].

Exosomes originate as ILVs within multivesicular bodies (MVBs), which subsequently fuse with the plasma membrane to release their vesicular contents [38,39]. The classical pathway relies on the endosomal sorting complex required for transport (ESCRT), comprising four subcomplexes (ESCRT-0, -I, -II, and -III) that act sequentially [40,41]. ESCRT-0 (HRS/STAM) recognizes ubiquitinated cargo via ubiquitin-binding domains while anchoring to endosomal phosphatidylinositol 3-phosphate. ESCRT-I and -II promote membrane budding; ESCRT-III, recruited by ESCRT-II or ALIX, drives vesicle scission. The ATPase *VPS4* then catalyzes membrane fission and ESCRT recycling [42,43].

While the ESCRT machinery plays a well-characterized role in protein and nucleic acid sorting, metabolite incorporation appears to involve distinct, lipid-dependent pathways [44]. Importantly, depletion of ESCRT machinery does not abolish exosome production, demonstrating that MVBs can also form through ESCRT-independent pathways [45,46]. The best-characterized ESCRT-independent mechanism involves neutral sphingomyelinase 2 (nSMase2; encoded by *SMPD3*), which hydrolyzes sphingomyelin to ceramide and phosphorylcholine [47,48]. Another study by [49] demonstrated that *SMPD3*-generated ceramide is required for sorting proteolipid protein into ILVs destined for secretion [49]. Ceramide induces negative membrane curvature, driving spontaneous budding and ILV formation [50,51]. Downstream, sphingosine-1-phosphate activates its receptor on MVBs to facilitate cargo segregation into ILVs [52]. Additional ESCRT-independent mechanisms include tetraspanin-enriched microdomains (*CD9*, *CD63*, *CD81*) and flotillin-mediated lipid raft-dependent ILV formation [53,54].

Solute carrier (SLC) transporters—a superfamily of over 450 transmembrane proteins spanning 65 families—serve as “metabolic gates,” mediating transport of glucose, amino acids, vitamins, neurotransmitters, and ions across cellular membranes [55,56,57]. The designation of SLCs as “metabolic gates” reflects their dual functionality: they actively concentrate specific metabolites into the ILV lumen during endosomal maturation, and subsequently mediate cargo release upon exosome fusion with recipient cell membranes. During ILV formation, SLCs oriented with their cytoplasm-facing domains exposed to the endosomal lumen can exploit the proton gradient maintained by vacuolar-type H^+^-ATPases (V-ATPases) within acidified MVBs, driving secondary active transport of metabolites into the nascent vesicle against their concentration gradients. This inward-directed transport phase is therefore energetically dependent on endosomal acidification and, indirectly, on the parent cell’s ATP availability. Upon exosome secretion and fusion with a recipient cell—either via plasma membrane merger or macropinosomal uptake—the reversal of electrochemical gradients across the recipient cell membrane can reactivate these same transporters in an outward-facing orientation, facilitating metabolite delivery into the recipient cell cytoplasm. Thus, SLC activity is not restricted to a single stage but represents a continuum of regulated transport events across the exosome lifecycle. Several SLC family members, including glucose transporters (*GLUT* family; *SLC2A* genes), amino acid transporters, and nucleoside transporters, have been identified in exosomal membranes [58]. Their activity is often coupled to electrochemical gradients or ATP hydrolysis, linking metabolite loading to the parent cell’s energetic status. Cryo-EM studies have revealed that SLCs adopt diverse folds—including major facilitator superfamily (MFS) and LeuT-like architectures—operating via rocker-switch or gated-pore mechanisms [59,60].

Exosomes are enriched in cholesterol, sphingolipids, phosphatidylserine, and ceramide, a composition resembling membrane lipid raft microdomains [26,28]. Lipid rafts contribute to protein sorting, membrane curvature, and vesicle budding, with raft components playing key roles in ESCRT-independent ILV formation [61]. Caveolin-1 and flotillins can drive lipid raft-dependent ILV formation, with the nSMase–ceramide pathway required in some cell lines [62]. Cholesterol levels across the plasma membrane, endoplasmic reticulum, and endosomal compartments influence both exosome content and size [63,64].

SLC transporter expression and activity are dynamically regulated in response to cellular metabolic state, creating a feedback loop whereby nutrient availability shapes exosomal metabolite composition [65,66]. SLCs are not only differentially but also dynamically expressed across cell types and tissues in response to environmental cues [67]. For instance, under glucose deprivation, enhanced exosome secretion and altered cargo loading have been demonstrated in cardiomyocytes, where exosomal protein and miRNA cargo shift in a glucose-dependent manner to modulate recipient cell function [68]. More broadly, cellular stresses including nutrient deprivation and hypoxia alter exosomal cargo composition [69]. In the tumor microenvironment, CAF-derived exosomes containing amino acids, lipids, and TCA cycle intermediates can rescue cancer cell proliferation under nutrient-deprived conditions, providing direct evidence that exosomes redistribute metabolic resources across cell populations [20]. This adaptive metabolite loading positions exosomes as metabolic buffers capable of intercellular nutrient redistribution [65,70].

Binding to luminal proteins offers an additional mechanism for metabolite retention. HSP90, a well-known exosome marker, can bind ATP and various small molecules, potentially serving as a metabolite chaperone within the vesicle lumen [71]. Proteomic studies have frequently identified metabolic enzymes—including glycolytic proteins and components associated with fatty acid metabolism—within extracellular vesicle preparations, suggesting a potential for continued metabolic activity within vesicles during transit [72,73]. Exosomes have been shown to generate extracellular ATP through glycolytic conversion, with glycolytic enzymes among the most commonly identified proteins in EV proteomics [74]. CAF-derived exosomes carry intact metabolite cargo—including amino acids, acetate, lactate, lipids, and TCA cycle intermediates—that recipient cancer cells directly utilize for central carbon metabolism [20,75]. However, it should be noted that high-resolution purification studies have challenged the presence of certain glycolytic enzymes (e.g., PKM, ENO1) in bona fide exosomes, suggesting some of these associations may reflect co-isolated non-vesicular material [76]. Nonetheless, the functional transfer of metabolites and metabolic enzymes via extracellular vesicles remains a compelling and active area of investigation, with implications for understanding exosomes as mobile metabolic compartments [77,78].

**Figure 2:**
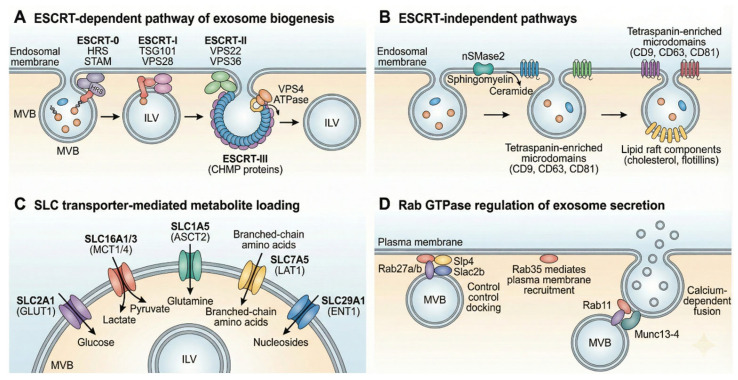
Molecular Mechanisms of Metabolite Encapsulation into Exosomes. (**A**) ESCRT-dependent pathway of exosome biogenesis. Sequential recruitment of ESCRT-0 (HRS, STAM), ESCRT-I (TSG101, VPS28), ESCRT-II (VPS22, VPS36), and ESCRT-III (CHMP proteins) to endosomal membranes mediates cargo recognition, membrane invagination, and intraluminal vesicle (ILV) formation. (**B**) ESCRT-independent pathways. Neutral sphingomyelinase 2 (nSMase2) generates ceramide from sphingomyelin, inducing membrane curvature and ILV budding. (**C**) SLC transporter-mediated metabolite loading. (**D**) Rab GTPase regulation of exosome secretion.

The secretion of exosomes requires trafficking of MVBs to the plasma membrane and subsequent membrane fusion. This process is orchestrated by Rab GTPase family members, particularly *RAB7*, *RAB11*, *RAB27A*, *RAB27B*, and *RAB35* [79,80,81]. *RAB27A* and *RAB27B* control different steps of the exosome secretion pathway: *RAB27A* mediates MVB docking, tethering, and fusion with the plasma membrane by interacting with effector proteins like Slp4, while *RAB27B* facilitates directed transfer of MVBs along microtubules to the actin-rich cortex (Figure 2) [82,83]. *RAB35* promotes MVB–plasma membrane docking and fusion in a CD2AP-dependent manner [80,84]. *RAB11* promotes calcium-dependent MVB–plasma membrane fusion by regulating vesicle docking/tethering [85]. A GTPase switch from Rab7 to Arl8b, facilitated by endoplasmic reticulum–MVB membrane contact sites, determines whether MVBs traffic toward lysosomes for degradation or toward the plasma membrane for exosome release [86]. SNARE proteins mediate the final membrane fusion event [87].

### 2.2. Metabolic State as a Determinant of Exosomal Cargo

The metabolic state of the parent cell profoundly influences the metabolite profile of secreted exosomes. Metabolic reprogramming is recognized as one of the hallmarks of cancer, representing an adaptive mechanism by which rapidly proliferating cancer cells adapt to increasing energy demands [88,89]. The Warburg effect, characterized by the preferential conversion of glucose to lactate even in the presence of oxygen and functional mitochondria, produces exosomes enriched in lactate, pyruvate, and glycolytic intermediates [90,91]. This metabolic phenotype, originally observed by Otto Warburg in 1924, supports rapid energy production and provides intermediates for biosynthesis of lipids, amino acids, and nucleotides required for cancer cell growth [92,93].

**Figure 3:**
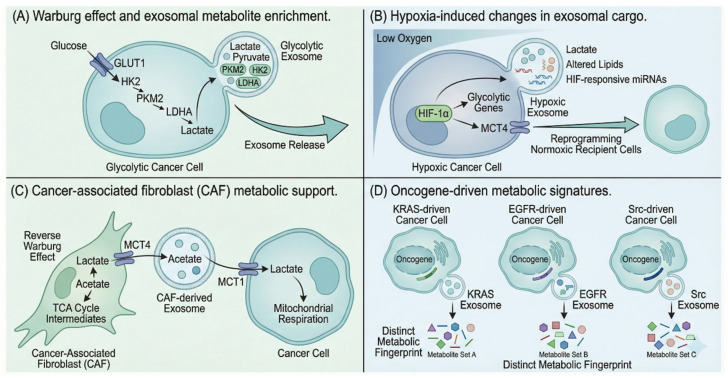
Metabolic state determines exosomal cargo composition. (**A**) Warburg effect and exosomal metabolite enrichment. Under aerobic glycolysis, cancer cells exhibit increased glucose uptake via GLUT1, elevated glycolytic flux through HK2 and PKM2, and lactate production via LDHA. (**B**) Hypoxia-induced changes in exosomal cargo. (**C**) Cancer-associated fibroblast (CAF) metabolic support. The “reverse Warburg effect”: CAF-derived exosomes provide lactate, acetate, and TCA cycle intermediates as metabolites to fuel cancer cell mitochondrial respiration. (**D**) Oncogene-driven metabolic signatures.

A landmark study by Zhao and colleagues demonstrated that cancer-associated fibroblast (CAF)-derived exosomes provide various metabolites—including amino acids, lipids, and TCA cycle intermediates—that are avidly utilized by cancer cells under nutrient deprivation or stress conditions [20]. They found that exosomes contain substantial concentrations of lactate, acetate, citrate, pyruvate, α-ketoglutarate, and fumarate, acting as “off-the-shelf” metabolite cargo that can contribute to the entire compendia of central carbon metabolism within recipient cancer cells [20]. This finding established that exosomes not only enhance the Warburg effect phenotype but actively supply metabolic substrates to support tumor growth (Figure 3).

Hypoxic conditions similarly alter exosomal metabolite content, enriching for metabolites associated with anaerobic metabolism [94,95]. Hypoxia, sensed primarily through hypoxia-inducible factors (HIFs; encoded by *HIF1A* and related genes), is a remarkable trait of the tumor microenvironment that drives metabolic reprogramming and more frequent cell communication [96]. *PKM2*, a glycolytic pyruvate kinase isoenzyme increased by hypoxia, can increase exosome secretion, exemplifying how metabolic reprogramming affects exosome biogenesis [97]. The composition of exosomes reflects the hypoxic status of tumor cells and can be used for tracking metabolic reprogramming [98]. Hypoxia upregulates or downregulates expression of key factors including non-coding RNAs, mRNAs, proteins, and lipids in hypoxic cells and their secreted exosomes [99].

Various factors, including cellular stress (hypoxia, nutrient deprivation, oxidative stress) and presence of oncogenes such as *SRC*, *EGFRvIII*, and *KRAS*, can impact exosomal cargo composition [17,69]. The presence of *SRC* increases release of exosomes containing proinflammatory proteins, while *KRAS* leads to increased exosomes containing proteins and miRNAs involved in cell growth and survival. Modulating signaling in exosome biogenesis pathways can affect contents, suggesting that regulating exosomal composition is a promising avenue for therapeutic development [100].

In HACE, prolonged hypobaric hypoxia alters the exosomal metabolome with significant downregulation of purine metabolites including adenosine and guanosine, reflecting disrupted energy metabolism [31]. This coupling between cellular metabolic state and exosomal metabolite composition enables exosomes to serve as metabolic messengers, transmitting real-time information about the metabolic environment of their cell of origin.

The “reverse Warburg effect” describes how cancer cells promote glycolysis in CAFs, which then provide metabolites for cancer cells, facilitating proliferation through the TCA cycle and oxidative phosphorylation (OXPHOS) [23]. The lactate symporters *MCT1* (*SLC16A1*) and *MCT4* (*SLC16A3*) are critical regulators in establishing a lactate shuttle system: *MCT4* favors export of lactate while *MCT1* facilitates cellular lactate uptake [101]. Exosomes mediate this metabolic crosstalk, with hypoxia inducing chemoresistant cells to secrete abundant exosomal *PKM2*, promoting glycolysis and production of reductive metabolites [102]. Exosomal *PKM2*-mediated metabolism reprogramming of CAFs results in a shift toward glycolysis, creating an acidic microenvironment that ultimately confers and maintains drug resistance [100].

Whether metabolite loading is predominantly selective or stochastic remains an open question. Evidence supports both mechanisms operating in parallel. The enrichment of certain metabolites relative to their cytosolic concentrations suggests active selection, possibly mediated by specific SLC transporters or binding proteins [103]. The presence of diverse metabolites at concentrations reflecting cytosolic levels indicates passive equilibration during ILV formation [36]. The relative contribution likely varies by metabolite class, cell type, and physiological context. Recent advances in single-vesicle analysis and isotope tracing approaches are beginning to address this fundamental question [11,104].

Emerging evidence suggests that specific post-translational modifications of metabolic enzymes can redirect their localization to the endosomal compartment, thereby influencing exosomal metabolite content. Acetylation of mitochondrial enzymes, a modification sensitive to cellular acetyl-CoA levels, has been implicated in regulating protein sorting to exosomes [105]. This mechanism creates a direct link between cellular acetyl-CoA pools—themselves reflective of overall energy status—and exosomal cargo composition. Similarly, hypoxia-induced SUMOylation of glycolytic enzymes may promote their packaging into exosomes, contributing to the metabolic adaptation of recipient cells within the hypoxic tumor microenvironment [106,107].

The distinction between selective and stochastic loading has important functional implications that deserve critical discussion. Passively or randomly loaded EV cargoes are defined as those that do not undergo local increases in concentration by clustering at EV biogenesis sites, meaning that if metabolite incorporation is largely stochastic, the metabolite composition of EVs would be expected to approximate the cytosolic metabolite concentrations of the donor cell, with no specific enrichment above cytosolic levels [104,108]. In this scenario, EVs would function as passive carriers of intracellular metabolites rather than as specialized signaling entities. Exosomes, nanoscale EVs secreted by all cells, carry bioactive cargo including proteins, nucleic acids, lipids, and metabolites—cargo that not only reflects cellular states but also actively regulates physiological and pathological processes [14]. However, demonstrating such active regulation requires evidence beyond mere presence. Importantly, if cytosolic concentrations are used as the loading baseline, the absolute amounts of any given metabolite per vesicle would be extremely small given the orders-of-magnitude smaller volume of an exosome relative to a typical mammalian cell [109]. Demonstrating a functional signaling role for EV metabolites therefore requires evidence of selective enrichment mechanisms resulting in vesicular metabolite concentrations exceeding those of the cytosol [108]. A shared stochastic pathway has been proposed to mediate exosome protein budding along both plasma and endosome membranes [110], suggesting that a proportion of vesicular cargo—including metabolites—may not be selectively sorted but rather reflects proximity and local concentration at the site of vesicle formation. While potential mechanisms such as SLC transporters, lipid raft partitioning [26], and metabolite binding to luminal proteins have been proposed, selective cargo loading is mediated by machinery such as the ESCRT complex [69], which creates patches of membrane with higher cargo concentrations that are then incorporated into budding EVs, though in many cases the adaptors for cytosolic cargoes remain less defined [111]. EV cargo composition differs depending upon the donor cell type, metabolic cues, and disease states, and unravelling how different cargoes are sorted into EVs in a regulated and context-specific manner is essential to understanding the specificity of EV-mediated signalling [110]. Direct experimental evidence supporting active sorting of specific metabolites into EVs remains limited, and while recent research has shown that the components of exosomal cargo are far more complicated than previously appreciated and do contain metabolites, most research continues to focus on exosomal proteins and RNAs rather than metabolites [14]. Available quantitative data on metabolite enrichment ratios—comparing vesicular to cytosolic concentrations—are therefore sparse and represent a priority for future investigation.

### 2.3. Quantitative Constraints on Exosomal Metabolite Delivery

A critical yet systematically underappreciated question in the exosomal metabolomics field concerns whether the quantities of metabolites packaged within extracellular vesicles (EVs) are sufficient to meaningfully perturb intracellular metabolite pools in recipient cells. A straightforward biophysical analysis reveals the magnitude of this challenge. An exosome of approximately 100 nm in diameter—consistent with the size range of small EVs as defined by the International Society for Extracellular Vesicles [11]—possesses an internal lumenal volume of roughly 5 × 10^−19^ L. This is approximately eight million-fold smaller than the cytoplasmic volume of a typical mammalian cell (~20 μm diameter, ~4 × 10^−12^ L). Even under the optimistic assumption that a given metabolite is present at millimolar concentrations within the vesicle lumen, this would correspond to no more than 300–3000 molecules per exosome. Accordingly, a simple mass-balance calculation demonstrates that elevating an intracellular metabolite concentration by as little as 10 μM in a recipient cell would necessitate the internalization and complete cargo release of approximately 10^4^–10^5^ exosomes per cell—conditions that assume quantitative cargo release and negligible metabolic turnover. Achieving a biologically substantial perturbation of 1 mM in a key metabolic pool would require on the order of 10^6^ or more vesicles per cell [112,113,114].

In practice, the effective number of vesicles required is likely to be considerably higher, for at least three reasons. First, a significant fraction of internalized EVs are routed through the canonical endo-lysosomal pathway—progressing from early endosomes to late endosomes and ultimately to lysosomes—where their cargo is subject to enzymatic degradation rather than productive release into the cytosol [115,116]. The endosomal escape efficiency of EV cargo is poorly characterized but is widely regarded as a rare event, analogous to the inefficient cytosolic delivery observed with lipid nanoparticles and other nanoscale carriers [117]. Second, metabolites that do reach the cytosol are subject to rapid consumption by ongoing metabolic reactions, reducing the steady-state accumulation of delivered substrates. Third, the actual lumenal metabolite concentrations in EVs remain poorly constrained; for many small molecules, passive or active packaging into nascent intraluminal vesicles may be less efficient than in the parent cytosol, yielding lumenal concentrations well below cytosolic levels.

These quantitative constraints carry direct mechanistic implications. In the majority of physiological contexts, EV-associated metabolites are more plausibly interpreted as acting through localized receptor-mediated signaling—for example, at the cell surface or within endosomal microcompartments—rather than as substantive sources of metabolic substrates capable of globally reprogramming the metabolome of recipient cells. The conceptual distinction between these two operational modes must be explicitly considered when interpreting experimental results. Canonical examples of the receptor-mediated mode include exosomal ATP activating P2 purinergic receptors [21,118,119], and sphingosine-1-phosphate (S1P) engaging cognate S1P receptors [19]; in both cases, trace quantities of a bioactive metabolite are sufficient to initiate robust downstream signaling cascades through well-characterized receptor-effector systems. By contrast, the proposed role of cancer-associated fibroblast (CAF)-derived exosomes in supplying carbon substrates—including lactate, acetate, amino acids, and TCA cycle intermediates—for anabolic and bioenergetic utilization by recipient cancer cells [20,120] invokes the bulk substrate delivery mode, which is inherently constrained by the quantitative arguments outlined above. Notably, even the landmark study employing ^13^C-stable isotope tracing to demonstrate the uptake of CDE-derived metabolites by prostate cancer cells was conducted under nutrient-depleted conditions, a caveat that is central to the mechanistic interpretation and that limits direct extrapolation to metabolite-replete physiological microenvironments [120].

To rigorously establish that EV-associated metabolites can meaningfully influence cellular metabolism through bulk delivery, future studies should satisfy a more stringent experimental framework. Specifically, such studies should include: (i) absolute quantification of individual metabolites within EV preparations, normalized to vesicle particle number as recommended by MISEV2023 [11] (ii) empirical measurement of the number of vesicles internalized per recipient cell under physiologically relevant dosing conditions; (iii) quantitative estimation of the fraction of EV cargo that successfully accesses the cytosol rather than remaining sequestered within endo-lysosomal compartments [115,117] (iv) direct comparison of the metabolite flux delivered via EVs against the endogenous metabolic flux of recipient cells using ^13^C- or other stable-isotope tracers [20] and (v) stringent controls to exclude contributions from co-isolated free metabolites or non-vesicular extracellular particles, including lipoproteins and protein aggregates, which are recognized confounders in EV preparations [11,118].

## 3. Signaling Mechanisms of Exosomal Metabolites

Beyond their established roles as energy substrates and biosynthetic precursors, metabolites encapsulated within exosomes can function as signaling molecules capable of eliciting significant biological responses in recipient cells. This emerging paradigm of metabolite-mediated intercellular communication draws on both established metabolite receptor biology and a growing body of exosome-specific experimental evidence. It is important to acknowledge that the strength of evidence varies considerably across metabolite classes, and many proposed functional outcomes have been inferred from intracellular biology rather than directly demonstrated through exosome-mediated transfer experiments.

Beyond their established roles as energy substrates and biosynthetic precursors, metabolites encapsulated within exosomes can function as signaling molecules capable of eliciting significant biological responses in recipient cells. This paradigm of metabolite-mediated intercellular communication represents an emerging frontier in cell biology; wherein small molecules historically viewed as passive metabolic intermediates are now recognized as active participants in cellular signaling networks [99,119,121]. It is important to acknowledge that the strength of evidence varies considerably across metabolite classes, and many proposed functional outcomes have been inferred from intracellular biology rather than directly demonstrated through exosome-mediated transfer experiments. The exosomal metabolome comprises a diverse repertoire of bioactive species, including nucleotides, tricarboxylic acid (TCA) cycle intermediates, lipid mediators, and redox cofactors, each capable of engaging specific receptor systems or modulating intracellular metabolic pathways to orchestrate recipient cell phenotypes [20,122]. Table 1 summarizes the major classes of exosomal metabolites and their signaling functions.

Metabolite-mediated exosomal signaling operates on a fundamentally different timescale than protein or nucleic acid cargo. While miRNA effects require hours to days to manifest through transcriptional changes, metabolites elicit functional responses within seconds to minutes of cellular uptake [20,78]. This distinction is critical in contexts demanding rapid physiological adaptation—acute hypoxia, exercise, or immune challenge. Consequently, exosomal metabolites may function as “first responders,” rapidly priming recipient cells before subsequent gene regulatory changes from nucleic acid cargo take effect [65]. This temporal advantage, however, applies primarily to receptor-mediated signaling by surface-exposed or quickly released EV metabolites (e.g., ATP, S1P, eicosanoids), not bulk substrate delivery, which remains subject to the quantitative constraints discussed in Section 2.3.

### 3.1. Direct Receptor Activation by Exosomal Metabolites

**Figure 4:**
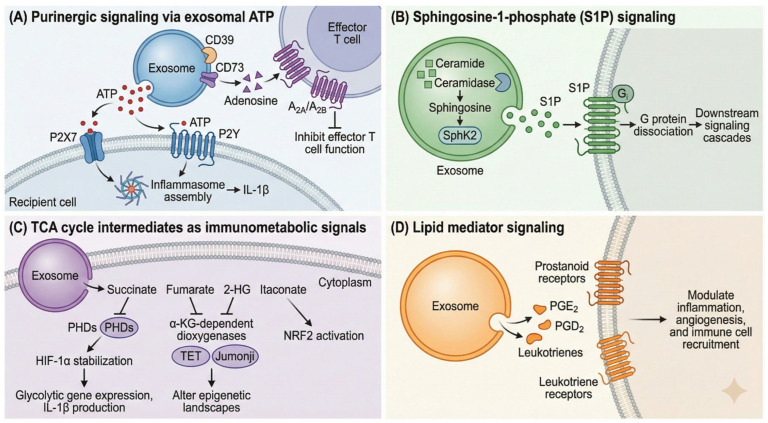
Signaling mechanisms of exosomal metabolites. Where indicated, evidence levels distinguish between direct exosomal transfer studies and established downstream pathway biology. (**A**) Purinergic signaling via exosomal ATP. Released ATP activates ionotropic P2X receptors (P2X1-7) and metabotropic P2Y receptors on recipient cells; ATP has been directly quantified in tumor-derived exosomes [118,135]. (**B**) Sphingosine-1-phosphate (S1P) signaling. Ceramide is converted to sphingosine by ceramidase, then phosphorylated by sphingosine kinase 2 (SphK2) to generate S1P; exosomal S1P has been demonstrated in several cancer cell lines [19,52]. (**C**) TCA cycle intermediates as immunometabolic signals; succinate detection in EVs has been reported [124], while downstream HIF-1α stabilization and macrophage polarization represent well-characterized cellular signaling pathways [125] that serve as plausible functional outcomes of exosomal succinate delivery. (**D**) Lipid mediator signaling via prostanoid and leukotriene receptors; phospholipases and prostaglandins have been detected in exosomal fractions [21,126].

Purinergic signaling represents one of the most extensively characterized pathways through which exosomal metabolites exert biological effects (Figure 4). Adenosine triphosphate (ATP), abundantly present in exosomal cargo, serves as a danger-associated molecular pattern (DAMP) that activates P2 purinergic receptors upon release into the extracellular milieu [118,132]. The P2 receptor family comprises ionotropic P2X receptors (P2X1-7), which function as ligand-gated cation channels, and metabotropic P2Y receptors, which couple to G-proteins to mediate diverse downstream signaling cascades [123,133]. Within the tumor microenvironment, extracellular ATP accumulates to concentrations sufficient to activate P2X7 receptors, triggering inflammasome assembly, interleukin-1β release, and modulation of anti-tumor T cell responses [134,135,136].

Recent investigations have identified P2RY2 as a critical purinergic immune checkpoint, wherein ATP-P2RY2 signaling drives cyclooxygenase-1/2 upregulation and accumulation of immunosuppressive prostaglandin E2 within solid tumors [137]. This mechanism provides a unifying explanation for pathological COX-PGE2 hyperactivation in the tumor microenvironment and has spurred interest in P2RY2 as a therapeutic target for combination immunotherapy. The ectonucleotidases CD39 and CD73, frequently expressed on tumor-derived and regulatory T cell-derived exosomes, sequentially hydrolyze ATP to adenosine monophosphate and subsequently to adenosine, generating an immunosuppressive adenosinergic milieu that inhibits effector T and natural killer cell functions while promoting regulatory T cell activity [138,139,140].

Sphingosine-1-phosphate (S1P), a bioactive lysophospholipid metabolite derived from ceramide catabolism, exemplifies the sophisticated signaling capacity of exosomal lipid mediators. S1P binds to a family of five G protein-coupled receptors (S1PR1-5) to regulate lymphocyte trafficking, vascular development, and immune homeostasis [19,141,142]. Intriguingly, S1P signaling at endosomal membranes is essential for exosome biogenesis itself; sphingosine kinase 2-mediated generation of S1P within multivesicular endosomes activates Gi-coupled S1P receptors, triggering G protein dissociation and cargo sorting into intraluminal vesicles [52]. This autocrine signaling loop positions S1P metabolism at the nexus of exosome production and function.

Exosomes also transport prostaglandins, leukotrienes, and other eicosanoid metabolites that engage prostanoid receptors on recipient cells to modulate inflammatory responses, angiogenesis, and tumor progression [21,126,143,144]. The transfer of bioactive lipids via exosomes enables donor cells to exert paracrine and endocrine effects on distant tissue sites, effectively extending the range of lipid mediator signaling beyond the local microenvironment. Moreover, exosome-mediated transfer of NDPK (nucleoside diphosphate kinase) facilitates ectopic expression of this phosphotransferase on recipient cell surfaces, where it generates nucleotide triphosphates to activate adjacent purinergic receptors, thereby creating a feedforward signaling circuit [145].

### 3.2. Immunometabolic Modulation via Exosomal TCA Cycle Intermediates

The recognition that TCA cycle intermediates function as signaling metabolites beyond their canonical biosynthetic roles has revolutionized understanding of metabolic regulation in immune cells. Succinate, fumarate, and α-ketoglutarate (α-KG) have emerged as potent immunomodulators that shape macrophage polarization, T cell function, and inflammatory responses through epigenetic and post-translational mechanisms [124,146,147]. These oncometabolites accumulate in cells harboring mutations in succinate dehydrogenase (SDH), fumarate hydratase (FH), or isocitrate dehydrogenase (IDH), and their presence in tumor-derived exosomes can reprogram recipient cell metabolism to favor tumor progression [148,149,150].

**Figure 5:**
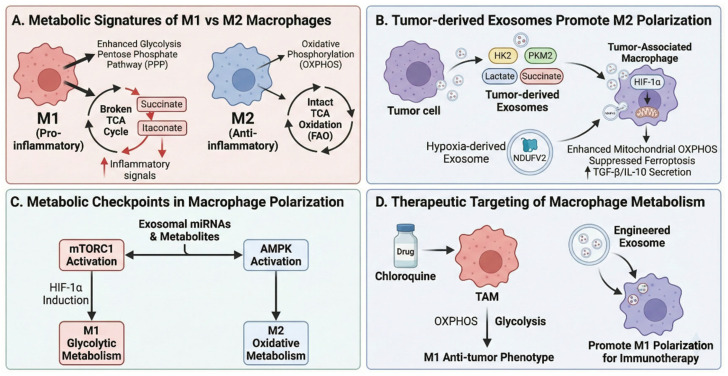
Exosome-mediated macrophage polarization and metabolic reprogramming. This figure integrates established macrophage immunometabolism with emerging evidence for exosome-mediated transfer; panels A and C depict well-characterized intracellular metabolic signatures, while panels B and D represent their proposed exosome-dependent modulation. (**A**) Metabolic signatures of M1 vs. M2 macrophages. M1 (pro-inflammatory) macrophages exhibit enhanced glycolysis and a “broken” TCA cycle with accumulation of succinate and itaconate [125,130]. M2 (anti-inflammatory) macrophages rely on oxidative phosphorylation (OXPHOS) [151]. The metabolite signatures depicted here represent intracellular polarization states; the contribution of exosomal metabolite delivery to establishing these states is a subject of active investigation [158,159]. (**B**) Tumor-derived exosomes promote M2 polarization; tumor exosomes enriched in lactate and TCA intermediates have been shown to skew macrophage polarization toward an anti-inflammatory phenotype [35,157]. (**C**) Metabolic checkpoints in macrophage polarization. (**D**) Therapeutic targeting of macrophage metabolism; pharmacological modulation of macrophage metabolic state via exosome-delivered metabolites represents an emerging strategy [155,156].

Macrophage polarization exemplifies the metabolic plasticity underlying immune cell function. Pro-inflammatory M1 macrophages rely predominantly on aerobic glycolysis and exhibit a broken TCA cycle characterized by accumulation of succinate and itaconate, whereas anti-inflammatory M2 macrophages utilize oxidative phosphorylation (OXPHOS) and intact TCA cycle flux fueled by fatty acid oxidation [151,152,153,154]. Succinate accumulation in M1 macrophages stabilizes hypoxia-inducible factor 1α (HIF-1α), which in turn activates glycolytic gene transcription and sustains interleukin-1β production [125]. Tumor-derived exosomes exploit these metabolic dependencies by delivering lactate, succinate, and glycolytic enzymes (hexokinase 2, pyruvate kinase M2) to tumor-associated macrophages, promoting their polarization toward immunosuppressive M2-like phenotypes (Figure 5) [155,156,157].

Recent single-cell metabolomics studies have revealed remarkable heterogeneity in macrophage metabolic states within tissues, suggesting that exosome-mediated metabolite transfer may serve to coordinate metabolic phenotypes across macrophage populations [158,159]. This “metabolic quorum sensing” mechanism could explain how spatially distributed immune cells achieve synchronized responses to tissue damage or infection. Moreover, the ability of tumor-derived exosomes to deliver oncometabolites such as 2-hydroxyglutarate (2-HG) to tumor-associated macrophages represents a novel mechanism of epigenetic reprogramming, as 2-HG competitively inhibits α-ketoglutarate-dependent dioxygenases including TET DNA demethylases and Jumonji-domain histone demethylases, leading to global changes in DNA and histone methylation patterns that favor immunosuppressive phenotypes [160].

Itaconate, produced from cis-aconitate by aconitate decarboxylase 1 (ACOD1/IRG1) in activated macrophages, has emerged as a key immunoregulatory metabolite with context-dependent effects on tumor immunity [130,131]. Itaconate inhibits SDH activity, activates the antioxidant transcription factor NRF2, and modifies proteins through cysteine succination, collectively modulating redox balance and inflammatory signaling [161,162]. The transfer of itaconate via exosomes enables systemic dissemination of these immunomodulatory effects. Fumarate, another TCA cycle-derived immunometabolite, regulates immune cell function through protein succination and inhibition of α-KG-dependent dioxygenases, including TET enzymes and Jumonji-domain histone demethylases, thereby altering epigenetic landscapes in recipient cells [163,164,165].

#### 3.2.1. Exosomal Metabolites in T Cell Function

T cell activation and differentiation are intimately linked to metabolic reprogramming, with naïve T cells primarily relying on oxidative phosphorylation for energy production, whereas effector T cells undergo a metabolic shift toward aerobic glycolysis to support rapid proliferation and enhanced cytokine production [166]. Tumor-derived exosomes significantly alter T cell metabolism through multiple distinct mechanisms within the immunosuppressive tumor microenvironment [167]. Exosomal delivery of adenosine and its precursors (AMP and ADP) activates adenosine A2A receptors on T cell surfaces, triggering cAMP-mediated suppression of T cell receptor (TCR) signaling and downstream effector functions. The extracellular adenosine pathway, generated through coordinated expression of exosomal ectonucleotidases CD39 and CD73, represents a critical mechanism of immune evasion, inhibiting effector T cell proliferation while simultaneously stimulating regulatory T cell (Treg) expansion and suppressive activity through A2a and A2b receptor signaling [167]. Lactate-enriched exosomes derived from metabolically active tumor cells directly acidify the local microenvironment and inhibit critical glycolytic enzymes, thereby impairing T cell proliferation and effector cytokine secretion. Additionally, exosomal transfer of kynurenine, a tryptophan metabolite generated by indoleamine 2,3-dioxygenase (IDO), activates aryl hydrocarbon receptor (AhR) signaling cascades to promote regulatory T cell differentiation while suppressing effector T cell responses. Kynurenine in IDO1-high cancer cell-derived extracellular vesicles promotes immunosuppression by inducing endothelial mitophagy in ovarian cancer, expanding the scope of exosome-mediated metabolic immunosuppression beyond T cell-intrinsic mechanisms [168].

The metabolic competition between tumor cells and infiltrating T lymphocytes for limiting nutrients—including glucose, glutamine, and arginine—represents an increasingly recognized determinant of anti-tumor immune responses. Altered tumor metabolic pathways inhibit cytotoxic T-cell function through multiple mechanisms, including glucose depletion, accumulation of lactic acid, and depletion of essential amino acids. Tumor-derived exosomes exacerbate this metabolic nutrient scarcity by sequestering nutrients within the tumor microenvironment or delivering metabolic enzymes that degrade essential amino acids. Recent evidence indicates that exosomal lipids, including sphingolipids such as sphingosine-1-phosphate (S1P), are linked to reduced cytotoxic T-cell activity, revealing an additional layer of lipid-mediated metabolic suppression [168]. Emerging therapeutic strategies involving engineered exosomes designed to deliver immune-stimulatory metabolites or inhibitors of immunosuppressive metabolic pathways represent promising approaches to restore T cell metabolic fitness within immunosuppressive tumor microenvironments. Technological advancements have allowed exosome modifications to magnify their intrinsic functions, load different therapeutic cargoes, and preferentially target tumor sites, with engineered exosomes exerting potent antitumor effects for cancer immunotherapy [169]. CAR-T cell-derived exosomes contain cytotoxic granules similar to their parent cells and have demonstrated significant anti-tumor activity in vitro and in animal models, suggesting that immune cell-derived exosomes may overcome limitations associated with direct cell therapy [170].

#### 3.2.2. Exosomal Metabolites in Cancer Metastasis

Cancer metastasis, the dissemination of tumor cells to distant organs, is increasingly recognized as a metabolically driven process in which exosomal metabolite signaling plays facilitating roles at multiple steps [143]. The metabolic remodeling of pre-metastatic niches—microenvironmental changes in secondary organs that precede tumor cell arrival—has been linked to exosome-mediated metabolite transfer. Tumor-derived exosomes enriched in S1P promote vascular permeability and immune suppression at distant sites, facilitating the homing of circulating tumor cells [141,142]. Exosomal lactate and succinate have been shown to induce M2-like macrophage polarization in the liver and lungs, establishing immunosuppressive pre-metastatic niches [23,125]. The Warburg-derived metabolites exported by exosomes from hypoxic primary tumors thus serve as advance metabolic signals that remodel distant tissues in preparation for metastatic colonization.

At the level of epithelial–mesenchymal transition (EMT), exosomal metabolites contribute to the phenotypic plasticity required for metastatic spread. Exosomal glutamine and branched-chain amino acids (BCAAs) promote mTORC1 activation and anabolic biosynthesis in recipient pre-metastatic cells [58]. Furthermore, exosomal 2-hydroxyglutarate (2-HG) from IDH-mutant tumors has been proposed to epigenetically reprogram stromal cells at distant sites through inhibition of α-KG-dependent demethylases, potentially contributing to niche preparation [160]. The exosomal metabolome thus functions as a metabolic “advance scout” that precedes metastatic dissemination, identifying it as a high-priority target for anti-metastatic therapeutic strategies.

### 3.3. Redox Regulation and Antioxidant Signaling

**Figure 6:**
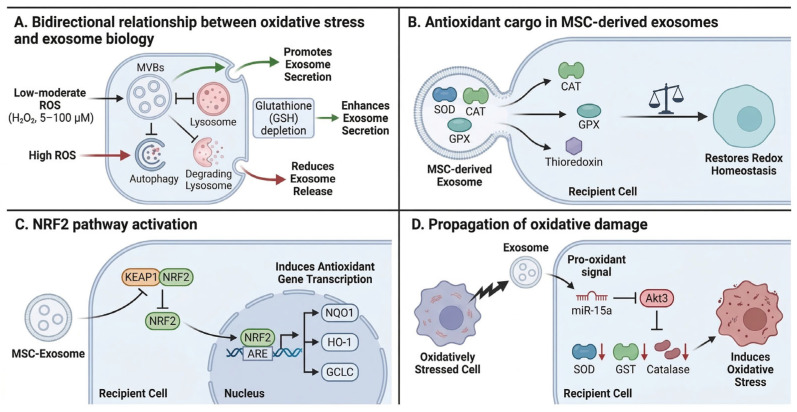
Redox regulation by exosomal metabolites and enzymes. (**A**) Bidirectional relationship between oxidative stress and exosome biology. Low-moderate ROS promotes exosome secretion by inhibiting lysosomal degradation of MVBs. (**B**) Antioxidant cargo in MSC-derived exosomes. Exosomes transfer superoxide dismutase (SOD), catalase (CAT), and glutathione peroxidase (GPX) to recipient cells. (**C**) NRF2 pathway activation. MSC-exosomes activate NRF2 signaling in recipient cells. (**D**) Propagation of oxidative damage through exosomes from oxidatively stressed cells.

Oxidative stress profoundly influences exosome biogenesis, composition, and function, establishing a bidirectional relationship between reactive oxygen species (ROS) and extracellular vesicle biology (Figure 6) [129,171,172]. Low to moderate concentrations of hydrogen peroxide promote exosome secretion in multiple cell types, while severe oxidative stress can attenuate exosome release through autophagy-mediated degradation of multivesicular bodies [97,173]. Intracellular ROS modulate exosome biogenesis by increasing MVB numbers through inhibition of lysosomal degradation, acting synergistically with depletion of exofacial glutathione (GSH) to enhance vesicle release [174]. 

Exosomes derived from healthy cells possess intrinsic antioxidant capacity, transferring superoxide dismutase (SOD), catalase, glutathione peroxidase (GPX), and other antioxidant enzymes to recipient cells experiencing oxidative damage [22,175,176]. Mesenchymal stem cell-derived exosomes activate the NRF2 signaling pathway in recipient cells, inducing expression of NAD(P)H quinone oxidoreductase 1 (NQO1), heme oxygenase 1 (HO-1), and other cytoprotective proteins that collectively restore redox homeostasis [128,177]. This mechanism underlies the therapeutic efficacy of MSC-derived exosomes in models of ischemia–reperfusion injury, oxidative stress-induced skin damage, and neurodegenerative diseases characterized by ferroptosis [178,179,180].

Conversely, exosomes from cells under oxidative stress can propagate redox imbalance to recipient cells. Exosomes isolated from irradiated mice induce oxidative damage in naive recipients, characterized by downregulation of SOD, glutathione-S-transferase, and catalase expression in splenic tissues [181]. Similarly, uroepithelial cell-derived exosomes generated under ketamine-induced oxidative stress activate P38-NF-κB signaling in recipient cells while suppressing NRF2 expression, thereby amplifying oxidative injury [182]. Plant-derived exosome-like nanovesicles represent an emerging source of exogenous antioxidants, containing high levels of ascorbic acid, glutathione, polyphenols, and flavonoids capable of restoring mitochondrial homeostasis and sirtuin expression in oxidatively stressed recipient cells [183,184].

## 4. Clinical Implications and Therapeutic Applications

### 4.1. Exosomal Metabolomics as Diagnostic Biomarkers

The unique metabolic signatures encapsulated within tumor-derived exosomes present unprecedented opportunities for non-invasive cancer detection through liquid biopsy approaches. Unlike tissue biopsies, which provide only spatially and temporally limited snapshots of tumor biology, circulating exosomes offer a minimally invasive window into real-time tumor metabolism that can be repeatedly sampled to monitor disease progression and therapeutic response [185,186,187]. The stability of exosomal membranes protects labile metabolites from degradation in circulation, preserving metabolic information that would otherwise be lost in cell-free plasma metabolomics approaches [1,188].

The integration of exosomal metabolomics with existing multi-omic approaches offers particular promise for early cancer detection [189]. Unlike genomic mutations, which may take years to accumulate, or protein biomarkers, which often lack specificity, metabolic alterations reflect real-time tumor biology and can precede clinical manifestation of disease [185]. Studies have demonstrated that exosomal metabolite signatures can detect pancreatic cancer and other malignancies earlier than conventional diagnostic methods, with prospective evidence suggesting detection capabilities extending up to two years before conventional diagnosis, highlighting the potential for metabolome-based screening in high-risk populations [190]. Furthermore, the metabolic signature of circulating exosomes can distinguish between primary and metastatic disease, providing valuable information for staging and treatment selection. The lipid bilayer nature of exosomes confers distinct advantages over other biomarkers, as the phospholipid membrane protects labile metabolites during sample handling and circulation, providing inherent stability superior to cell-free metabolomics approaches [190].

The concept of “metabolic liquid biopsy” extends beyond cancer to encompass metabolic diseases, neurological disorders, and cardiovascular conditions. Exosomal metabolites from adipose tissue-derived extracellular vesicles correlate with insulin resistance and can predict type 2 diabetes (T2DM) progression. Brain-derived exosomes crossing the blood–brain barrier carry metabolic signatures that distinguish between neurodegenerative diseases, potentially enabling non-invasive differential diagnosis of Alzheimer’s disease, Parkinson’s disease, and other dementias through analysis of metabolites such as glycerophosphocholine and other altered metabolic biomarkers [191]. This approach represents a paradigm shift in precision diagnostics, as exosomal metabolomic profiling captures the dynamic metabolic state of tissue-specific cells without requiring invasive procedures. Machine learning integration of metabolomic, proteomic, and transcriptomic data from exosomes further enhances diagnostic accuracy and enables stratification of disease subtypes, positioning exosome-based metabolomics as a cornerstone technology for next-generation precision medicine.

Mass spectrometry-based metabolomic profiling of tumor-derived exosomes has revealed distinct metabolic fingerprints capable of distinguishing cancer patients from healthy individuals and patients with benign lesions [30,192,193]. Liquid chromatography-tandem mass spectrometry (LC-MS/MS) and gas chromatography-mass spectrometry (GC-MS) enable comprehensive characterization of exosomal metabolomes, identifying alterations in glycolytic intermediates, amino acids, nucleotides, and lipids that reflect the metabolic reprogramming characteristic of malignancy [194,195]. Novel platforms combining magnetic nanoparticle-based exosome isolation with laser desorption/ionization mass spectrometry enable rapid metabolic fingerprinting for cancer diagnosis, as demonstrated in endometrial cancer screening [196].

Urinary extracellular vesicle metabolomics represents a particularly attractive non-invasive approach for early cancer detection. Metabolomic analysis of urinary EVs from lung cancer patients has identified distinct profiles of organic acids, lipids, and organoheterocyclic compounds that discriminate early-stage disease from healthy controls with high sensitivity and specificity [197]. Multi-omic integration combining exosomal proteomics, transcriptomics, metabolomics, and lipidomics further enhances biomarker discovery by providing comprehensive molecular portraits of tumor biology [18,198]. Artificial intelligence and machine learning algorithms applied to these complex datasets enable identification of robust multi-analyte signatures for personalized cancer diagnostics [199].

### 4.2. Engineered Exosomes as Therapeutic Delivery Vehicles

The intrinsic properties of exosomes—including biocompatibility, low immunogenicity, ability to cross biological barriers, and capacity for cellular targeting—position them as ideal candidates for therapeutic drug delivery [200,201,202]. Unlike synthetic nanoparticles that rely primarily on passive targeting through the enhanced permeability and retention (EPR) effect, exosomes can actively engage specific cellular receptors, improving delivery efficiency while minimizing off-target effects [203,204]. Clinical trials are currently evaluating exosome-based delivery of chemotherapeutic agents, including paclitaxel and doxorubicin, in drug-resistant breast and ovarian cancers [205].

Engineering strategies have substantially expanded the therapeutic potential of exosomes. Surface modification through genetic engineering, metabolic engineering, or direct membrane engineering of parent cells enables functionalization with targeting ligands that home to tumor-specific receptors such as EGFR, HER2, or PSMA [206,207,208]. Cargo loading can be achieved through endogenous (pre-secretory) approaches, wherein therapeutic agents are loaded into donor cells prior to exosome secretion, or exogenous (post-secretory) methods including electroporation, sonication, and incubation with permeabilizing agents [209,210]. Mesenchymal stem cell-derived exosomes loaded with gemcitabine have demonstrated enhanced apoptosis induction in pancreatic cancer models [211].

Exosome-mediated delivery of nucleic acid therapeutics, including small interfering RNAs (siRNAs), microRNAs, and CRISPR/Cas9 systems, offers promising approaches to overcome drug resistance mechanisms [212,213]. Exosomes loaded with miRNAs targeting the PI3K/AKT/mTOR pathway have reversed resistance to PI3K inhibitors in preclinical models, while CRISPR/Cas9 delivery targeting mutant KRAS G12C has restored temozolomide sensitivity in glioblastoma [214,215]. The cargo flexibility of exosomes, combined with their ability to deliver payloads directly into the cytoplasm of recipient cells, provides significant advantages over conventional delivery methods constrained by poor intracellular uptake or rapid drug degradation [24].

### 4.3. Metabolic Reprogramming as a Therapeutic Target

Understanding exosome-mediated metabolic communication in the tumor microenvironment reveals novel therapeutic opportunities for disrupting tumor-stroma metabolic symbiosis. Targeting exosome biogenesis pathways, including inhibition of nSMase2 with agents such as GW4869, can attenuate tumor-derived exosome secretion and impair metabolic reprogramming of stromal cells [216,217]. However, broad-spectrum exosome inhibition simultaneously blocks both tumor-promoting and immune-stimulatory vesicle transport, highlighting the need for more selective targeting strategies [218].

Purinergic signaling pathways represent attractive therapeutic targets given the profound immunomodulatory effects of exosomal ATP and adenosine. *ENTPD1* (CD39) and *NT5E* (CD73) inhibitors are under clinical investigation to disrupt adenosinergic immunosuppression in solid tumors, potentially synergizing with immune checkpoint blockade [219,220]. P2X7 receptor antagonists and P2RY2 inhibitors offer complementary approaches to modulate ATP-driven immune evasion and PGE2 accumulation in the tumor microenvironment [40,221]. The concept of “purinergic immune checkpoints” has emerged as a unifying framework for developing combination immunotherapies targeting exosome-mediated metabolic communication [221]. Table 2 summarizes the current clinical applications and therapeutic strategies targeting exosomal metabolic communication.

### 4.4. Exosomal Metabolite Signaling in Viral Infections

The role of exosomal metabolites in viral pathogenesis and host defense has emerged as an important frontier in extracellular vesicle biology. Viral infections profoundly remodel host cell metabolism, and these metabolic changes are reflected in the composition of secreted exosomes. Virus-infected cells release exosomes with altered metabolite cargo that can reprogram recipient immune cells, modulate antiviral responses, and facilitate viral spread [17].

HIV-infected macrophages release exosomes enriched in glycolytic metabolites and NAD+ precursors that suppress CD8+ T cell cytotoxicity through activation of the adenosinergic pathway [138,139]. SARS-CoV-2 infection dramatically alters cellular metabolomics, enriching exosomes with succinate and itaconate—metabolites that modulate NF-κB signaling and type I interferon responses in recipient cells [130,131]. Succinate-enriched exosomes from COVID-19 patients have been reported to activate HIF-1α in endothelial cells, potentially contributing to the vascular inflammation characteristic of severe disease. The biological “intent” of this succinate-enriched exosomal signaling warrants consideration. Two mechanistically distinct interpretations exist: (i) a host-defense scenario, in which succinate-loaded exosomes serve as paracrine danger signals that alert neighboring uninfected cells to the metabolic disturbance caused by viral infection, potentially priming innate immune responses; or (ii) a viral-manipulation scenario, in which SARS-CoV-2 co-opts host exosome biogenesis to propagate succinate-driven HIF-1α and NF-κB activation, thereby inducing the vascular inflammation and cytokine storm that exacerbate disease severity. Current evidence does not decisively distinguish between these possibilities, as both are consistent with the observed HIF-1α activation in recipient endothelial cells. However, the temporal dynamics of exosomal succinate enrichment relative to viral replication kinetics, and the known role of succinate-SUCNR1 signaling as an amplifier of vascular inflammation, are more consistent with the viral-manipulation interpretation in severe COVID-19. Future studies employing cell-type-specific exosome depletion strategies will be essential to resolve this question.

Conversely, exosomal metabolites can also serve antiviral functions. Plant-derived extracellular vesicles rich in antioxidant metabolites have demonstrated anti-inflammatory activity relevant to viral tissue damage [183]. Exosomal NAD+ transfer has been proposed as a mechanism by which healthy cells communicate metabolic sufficiency to virus-challenged neighbors, potentially coordinating tissue-level antiviral responses [74]. As the exosomal metabolomics field matures, characterization of infection-specific metabolite signatures holds promise for developing novel diagnostic biomarkers and metabolite-based antiviral strategies.

### 4.5. Exosomal Metabolites in Metabolic Diseases

Exosomal metabolites have emerged as important mediators of inter-organ metabolic crosstalk in type 2 diabetes mellitus (T2DM) and non-alcoholic fatty liver disease (NAFLD), two of the most prevalent metabolic disorders globally. In T2DM, adipose tissue-derived exosomes carry elevated levels of branched-chain amino acids (BCAAs), ceramides, and lysophosphatidylcholines that impair insulin signaling in skeletal muscle and hepatocytes [15,16,222,223]. Adipocyte-derived exosomes from insulin-resistant donors activate the mTORC1 pathway in recipient cells and suppress IRS-1 phosphorylation, mechanistically linking exosomal metabolite cargo to peripheral insulin resistance [223]. Furthermore, circulating exosomal metabolite signatures including altered acylcarnitine profiles and nucleotide metabolites have been proposed as early biomarkers capable of predicting T2DM progression before clinical onset [15,16,224].

In NAFLD, hepatocyte-derived exosomes are enriched in sphingosine-1-phosphate (S1P), ceramides, and lysophosphatidic acid under lipotoxic conditions, and have been shown to activate hepatic stellate cells, promoting fibrosis progression [15,16,225]. Exosomal metabolomics of NAFLD patient plasma reveals consistent upregulation of TCA cycle intermediates (succinate, fumarate), free fatty acid derivatives, and bile acid conjugates relative to healthy controls, reflecting the perturbed mitochondrial and lipid metabolism characteristic of NAFLD [226]. These circulating exosomal metabolite signatures correlate with histological disease severity, suggesting their potential utility as non-invasive biomarkers for NAFLD staging. Importantly, gut microbiome-derived short-chain fatty acids (SCFAs), including butyrate and propionate, have been detected in gut epithelium-derived exosomes and may represent an additional route through which microbial metabolites influence hepatic lipid metabolism in NAFLD via the gut–liver axis [227].

Beyond T2DM and NAFLD, exosomal metabolite signaling has been implicated in obesity-related inter-organ crosstalk. Lipid-enriched exosomes from hypertrophic adipocytes impair pancreatic beta-cell function by delivering ceramides and oxidized lipids that trigger mitochondrial dysfunction and ER stress [15,16,222]. In skeletal muscle, exosomal lactate and succinate released during exercise mediate adaptive metabolic responses in distant tissues, providing a molecular basis for exercise-induced systemic metabolic benefits [228,229]. These examples collectively establish exosomal metabolomics as a promising diagnostic and mechanistic framework for understanding the systemic dysregulation characteristic of cardiometabolic disease.

### 4.6. Methodological Limitations and Standardization Challenges in Exosomal Metabolomics

Exosomal metabolomics faces a layered set of methodological challenges—spanning sample integrity, analytical sensitivity, isolation bias, and the absence of field-wide standards—that must be systematically addressed before clinical translation can be realized [230].

#### 4.6.1. Isolation Artifacts, Contamination, and Method-Dependent Bias

A foundational challenge in EV metabolomics is distinguishing genuinely encapsulated metabolites from those introduced through co-isolation artifacts, a problem compounded by the fact that the choice of isolation method itself systematically shapes the apparent metabolome of any EV preparation.

Three principal contamination sources warrant attention. Lipoproteins—particularly ApoB-100 (LDL) and ApoA-I (HDL)—overlap substantially with small EVs in size and buoyant density and are routinely co-isolated by most standard enrichment protocols, including differential ultracentrifugation (dUC), size exclusion chromatography (SEC), and polymer-based precipitation [231,232]. In plasma, lipoprotein concentrations exceed EV concentrations by five to six orders of magnitude [233], meaning even trace lipoprotein carryover constitutes a significant source of metabolite background signal. Non-vesicular extracellular nanoparticles (NVEPs)—including exomeres and supermeres—harbor metabolite and protein cargo previously attributed to canonical exosomes, and their inadvertent inclusion in EV preparations can substantially distort metabolomic profiles [234]. Finally, residual free metabolites from conditioned medium or biofluid incompletely removed during washing steps may co-sediment with vesicles, particularly for hydrophilic analytes such as nucleotides, amino acids, and TCA cycle intermediates.

These contamination risks are not equivalent across isolation methods, and method-dependent biases carry direct consequences for the interpretation of published datasets. Differential ultracentrifugation, while historically the field’s benchmark, co-isolates protein aggregates and lipoprotein-associated lipids that artificially inflate apparent metabolite content; pelleting forces also promote vesicle aggregation and membrane disruption that may release luminal contents prior to extraction [232,233]. SEC substantially improves purity by separating EVs from free-soluble metabolites and reducing NVEP contamination, but yields lower total metabolite recovery. Polymer-based precipitation kits are operationally simple and high-throughput but co-precipitate lipoproteins, protein complexes, and nucleoprotein aggregates, rendering metabolite data from these protocols particularly prone to misinterpretation. Immunoaffinity capture using tetraspanin-targeted antibodies (CD9, CD63, CD81) offers the greatest subpopulation specificity and effectively excludes lipoproteins—which lack tetraspanin expression—but remains limited in throughput, scale, and metabolite yield [232,233]. Certain metabolite classes are disproportionately affected by these method-specific biases: polar lipids and phospholipids are enriched in lipoprotein co-isolates; amino acids and organic acids tend to co-precipitate with protein aggregates; and purine nucleotides may adsorb non-specifically to EV membrane surfaces during prolonged ultracentrifugation. These patterns mean that cross-study comparisons of EV metabolite profiles are only meaningful when identical isolation protocols have been used.

Validation of true encapsulation requires a multi-pronged approach. Density gradient fractionation using iodixanol (OptiPrep) gradients remains the most reliable strategy for separating bona fide small EVs (buoyant density ~1.13–1.19 g/mL) from co-isolated LDL (~1.02 g/mL) and HDL (~1.08–1.21 g/mL), with co-fractionation alongside established EV markers (CD63, CD9, CD81, TSG101) serving as strong supporting evidence for genuine vesicular association [11,235]. Detergent lysis controls comparing metabolite release from intact versus permeabilized EVs can distinguish luminal encapsulation from surface adsorption [236], while proteinase K digestion prior to lysis further discriminates between outer membrane-associated and truly intraluminal metabolites. Immunoaffinity capture using tetraspanin-targeted antibodies provides an additional layer of specificity by enriching defined EV subpopulations while simultaneously excluding lipoproteins from downstream metabolite analysis [235,237].

Preparation purity should be assessed by the ratio of EV particle number to co-isolated lipoprotein or protein content, verified using lipoprotein-specific markers such as LDL and HDL alongside nanoparticle tracking analysis. Although density gradient ultracentrifugation represents the current gold standard for establishing genuine EV encapsulation, it is time-intensive and low-throughput, limiting routine application in large clinical cohort studies. The field therefore requires development and validation of scalable, high-purity isolation methods compatible with metabolomics workflows, a priority that should be explicitly addressed in future method-development studies and reflected in emerging EV metabolomics reporting guidelines.

#### 4.6.2. Analytical Sensitivity and Metabolite Stability

Compounding the contamination problem is a persistent analytical challenge: the quantity of metabolites recoverable from EV isolates frequently approaches the detection threshold of conventional mass spectrometry platforms [230]. This has driven interest in higher-sensitivity separation technologies, including capillary electrophoresis-mass spectrometry (CE-MS)—particularly suited to ionic and highly polar analytes such as nucleotides, organic acids, and amino acids that are poorly retained by reversed-phase LC columns—and microfluidic nano-liquid chromatography systems capable of operating at the picomole scale [238,239]. Advanced platforms such as LC-MS/MS and ion-mobility mass spectrometry (IM-MS) have meaningfully expanded metabolite coverage and profiling capacity; however, even with these improvements, detection of low-abundance EV metabolites remains technically demanding, and substantial sensitivity gains are still required [236,240].

Metabolite stability during processing constitutes a further confounding variable. Labile species including ATP, NADH, and eicosanoid lipid mediators can degrade substantially during EV isolation procedures, generating artifactual results that compromise biological interpretation and reproducibility across laboratories [235,238]. These stability concerns extend beyond the laboratory: once exosomes are released into the extracellular space, their phospholipid bilayer provides only partial protection against degradation, and signaling-competent half-lives vary substantially by metabolite class. Purine nucleotides (ATP, ADP) are rapidly hydrolyzed by extracellular ectonucleotidases (CD39, CD73) with half-lives on the order of minutes in tissue microenvironments [119,241], whereas eicosanoids and sphingosine-1-phosphate are susceptible to oxidative degradation and lipase activity over hours [141,242]. Redox cofactors such as NADH are particularly labile at physiological temperatures due to spontaneous hydrolysis and oxidation [243], while amino acids and TCA cycle intermediates are chemically more stable and may retain signaling competence over longer timescales. These differential stabilities have direct implications for the spatiotemporal range of exosomal metabolite signaling: receptor-active metabolites with short extracellular half-lives are likely to act in a paracrine or juxtacrine manner [119,141], whereas chemically stable metabolites may support more distal endocrine-type communication. Future studies should incorporate rigorous time-course measurements of metabolite integrity post-secretion, alongside stable isotope-labeled internal standards spiked prior to isolation, to accurately model the biologically available fraction of exosomal cargo.

#### 4.6.3. Standardization and the Path to Clinical Translation

The absence of harmonized pre-analytical and analytical protocols remains one of the most consequential barriers to cross-study comparability and clinical application [230,234,235]. While the MISEV2023 consensus guidelines provide the most current framework for EV isolation rigor, reproducibility, and transparency, metabolomics-specific recommendations within this document remain limited and represent an area requiring dedicated expert consensus [11]. Equally pressing is the lack of certified reference materials and validated inter-laboratory ring trials for EV metabolomics: unlike other omics disciplines, the field currently lacks batch-characterized reference specimens with defined metabolite cargo, creating a significant obstacle to method validation and assay benchmarking [235]. Addressing this gap will require the community to develop minimum reporting standards analogous to MIAPE (Minimum Information About a Proteomics Experiment) specifically tailored to exosomal metabolomics—encompassing isolation method, lysis and extraction protocol, analytical platform, and metabolite annotation criteria—to ensure that findings are reproducible, comparable across laboratories, and ultimately translatable into validated clinical assays [230,244]. As a concrete “call to action,” we propose that exosomal metabolomics studies adopt a minimum set of reporting requirements modeled on the MISEV2023 framework but extended to metabolomics-specific needs. Specifically, we recommend: (i) inclusion of a “blank media control” processed identically to the sample through all isolation steps, to quantify background metabolite carryover from conditioned medium; (ii) use of stable isotope-labeled recovery internal standards (e.g., ^13^C- or deuterium-labeled versions of key analytes) spiked prior to lysis, to correct for variable extraction efficiency and instrument response; (iii) reporting of metabolite concentrations normalized to both protein content and particle number, enabling cross-study comparisons; and (iv) mandatory disclosure of isolation method, lysis buffer composition, extraction solvent, and MS platform with acquisition parameters. Implementation of these four standards would represent a meaningful and immediately achievable improvement in the reproducibility and interpretability of exosomal metabolomics datasets.

## 5. Conclusions and Future Perspectives

The exosomal metabolome represents an important and functionally distinct intercellular signaling system coordinating metabolic states across distant cell populations. However, a nuanced view is warranted: the mode of action of EV metabolites likely differs substantially depending on the metabolite class and biological context. For receptor-active metabolites such as ATP, S1P, and eicosanoids, even small amounts delivered by EVs may elicit potent downstream signaling through receptor amplification cascades, supporting a genuine signaling role. For metabolites proposed to function as bulk substrates (TCA intermediates, amino acids, glycolytic products), the quantitative constraints discussed herein indicate that rigorous experimental demonstration is required before functional conclusions can be drawn.

Critical questions remain regarding metabolite packaging selectivity. Whether encapsulation is stochastic or involves active sorting mechanisms remains incompletely understood. Resolving this question is not merely academic: if loading is predominantly stochastic, the vesicular metabolite concentrations will approximate cytosolic levels, further compounding the quantitative delivery constraints. Conversely, evidence for active concentration of specific metabolites into EVs would suggest the existence of dedicated metabolite packaging machinery and would strengthen the case for functional metabolite transfer. Single-vesicle metabolomics and advanced microfluidic platforms promise unprecedented insights into exosome heterogeneity and real-time metabolite transfer dynamics.

The convergence with immunometabolism presents exciting therapeutic opportunities. Designer exosomes loaded with specific metabolite cocktails could transform treatment of cancer, autoimmunity, and metabolic disorders. Translation faces significant challenges including standardization of isolation methods, rigorous contamination controls, GMP-grade production protocols, and regulatory classification. Nevertheless, the versatility of exosomes as biomarkers, drug delivery vehicles, and therapeutic targets suggests exosomal metabolomics will assume a central role in precision medicine approaches to cancer and metabolic diseases. The concept of “Metabolic Quorum Sensing,” introduced in Section 3.2, deserves particular emphasis as a unifying conceptual framework for the field. Just as bacteria use diffusible small molecules to coordinate population-level behaviors, the evidence reviewed here supports the model that cells use exosomal metabolites to sense and synchronize metabolic states across tissues. Succinate, ATP, and S1P signaling via exosomes can be understood as components of a tissue-level metabolic quorum—a system that allows spatially distributed cell populations to achieve coordinated responses to nutrient stress, hypoxia, or infection without direct cell-to-cell contact. This framework provides a compelling rationale for why exosomal metabolomics matters beyond individual cell biology: it is a language of tissue-level metabolic coordination with broad implications for understanding homeostasis and disease. To provide a balanced perspective, it is important to acknowledge that not all proposed instances of exosomal metabolite transfer have yielded clear functional phenotypes in recipient cells. Several studies have reported that EVs carry detectable metabolite cargo without demonstrating measurable changes in recipient cell metabolism upon EV uptake, particularly when physiologically relevant EV doses and nutrient-replete conditions are employed. These null or inconclusive findings are not failures of the hypothesis per se, but rather they delineate the conditions under which metabolite transfer is functionally significant—namely, high local EV concentrations, metabolite-depleted microenvironments, and receptor-active cargo—from those where it is not. Acknowledging these limitations strengthens rather than weakens the scientific rigor of the field, and will help focus future efforts on the subset of exosomal metabolite interactions most likely to be biologically consequential.

## Figures and Tables

**Table 1:** Exosomal metabolites and their signaling functions.

Metabolite Class	Representative Metabolites	Receptor/Target	Biological Effects	Evidence Level	References
Purine nucleotides	ATP, ADP, AMP, Adenosine	P2X1-7, P2Y1/2/4/6/11-14, A1/A2A/A2B/A3	Inflammasome activation, T cell modulation, immunosuppression, angiogenesis	Direct EV detection (MS)	[18,123]
Sphingolipids	S1P, Ceramide, Sphingosine	S1PR1-5 (GPCRs)	Lymphocyte egress, vascular integrity, exosome biogenesis regulation	Direct EV detection (MS, lipidomics)	[19,52]
TCA cycle intermediates	Succinate, Fumarate, α-KG, Citrate	SUCNR1 (GPR91), PHDs, TETs, JmjC demethylases	HIF-1α stabilization, epigenetic reprogramming, macrophage polarization	Partial: succinate detected in EVs; fumarate/α-KG inferred from parent-cell biology	[124,125]
Glycolytic metabolites	Lactate, Pyruvate, Glucose	MCT1/4, GLUT1, GPR81 (lactate receptor)	Metabolic fuel supply, Warburg effect propagation, lactylation	Direct detection (NMR, MS)	[20,101]
Eicosanoids	PGE2, PGD2, LTB4, LTC4	EP1-4, DP1/2, BLT1/2, CysLT1/2	Inflammation, vasodilation, immune cell chemotaxis, tumor promotion	Direct EV detection (lipidomics)	[21,126]
Amino acids	Glutamine, Glutamate, Arginine, BCAAs	SLC1A5, SLC7A5, mTORC1 pathway	Anaplerosis, protein synthesis, mTOR activation, NO production	Partial: SLC transporter evidence; direct exosomal quantification limited	[58,127]
Redox cofactors	NAD+/NADH, GSH/GSSG, FAD	Sirtuins, PARPs, NRF2-KEAP1 pathway	Redox homeostasis, epigenetic regulation, antioxidant defense	Inferred: based on antioxidant enzyme cargo; direct metabolite detection limited	[128,129]
Immunometabolites	Itaconate, 2-HG	KEAP1, SDH, αKGDDs	Anti-inflammatory response, NRF2 activation, metabolic enzyme inhibition	Partial: 2-HG detected in cancer-derived EVs; itaconate inferred from ACOD1 activity	[130,131]

Abbreviations: S1P, sphingosine-1-phosphate; α-KG, α-ketoglutarate; 2-HG, 2-hydroxyglutarate; PHD, prolyl hydroxylase domain; TET, ten-eleven translocation; JmjC, Jumonji C domain; MCT, monocarboxylate transporter; GLUT, glucose transporter; PG, prostaglandin; LT, leukotriene; BCAA, branched-chain amino acid; GSH, glutathione; GSSG, glutathione disulfide; PARP, poly (ADP-ribose) polymerase; SDH, succinate dehydrogenase; αKGDD, α-ketoglutarate-dependent dioxygenase.

**Table 2:** Clinical applications and therapeutic strategies targeting exosomal metabolic communication.

Application	Approach/Agent	Mechanism	Clinical Status/Evidence	Key Metabolites/Pathways	References
Cancer detection	Exosomal metabolic fingerprinting (LC-MS/MS)	Altered glycolytic, amino acid, lipid metabolites	Preclinical validation; AUC 0.85–0.95	Altered amino acids, lipids, organic acids; glycolytic metabolites	[30,194]
Early detection	Urinary EV metabolomics	Non-invasive sampling; tumor metabolite enrichment	Stage I lung cancer validated	Organic acids, lipids, organoheterocyclic compounds	[186,197]
Treatment monitoring	Serial exosome metabolomics	Dynamic metabolic changes reflect therapy response	Under investigation	Dynamic metabolite flux (lactate, TCA intermediates)	[29,187]
Chemotherapy delivery	Exosome-encapsulated paclitaxel, doxorubicin	Enhanced tumor accumulation; reduced toxicity	Phase I/II trials	Paclitaxel, doxorubicin (drug loading, not endogenous metabolites)	[200,211]
Gene therapy	siRNA/CRISPR-loaded exosomes	Knockdown of resistance genes	Preclinical efficacy	siRNA (nucleic acid, not metabolite)	[212,213]
Targeted delivery	Surface-engineered exosomes	Receptor-mediated tumor homing	4–10 × improved delivery	Surface-modified lipids; EGFR/HER2 ligands	[207,208]
Exosome inhibition	GW4869 (nSMase2 inhibitor)	Blocks ceramide-mediated exosome formation	Preclinical	Ceramide (nSMase2 substrate)	[216,217]
Adenosinergic pathway	CD39/CD73 inhibitors	Blocks ATP → adenosine; restores T cell function	Phase I/II trials	ATP → Adenosine (CD39/CD73 axis)	[219,220]
Purinergic checkpoint	P2X7/P2RY2 antagonists	Blocks ATP-driven immune evasion	Preclinical validation	ATP (P2X7/P2RY2 ligand)	[137,221]
Macrophage repolarization	Chloroquine, metabolic modulators	Switches TAM from OXPHOS to glycolysis	Drug repositioning	Lactate, TCA intermediates (macrophage metabolic modulators)	[152,155]

Abbreviations: LC-MS/MS, liquid chromatography-tandem mass spectrometry; EV, extracellular vesicle; AUC, area under the curve; siRNA, small interfering RNA; CRISPR, clustered regularly interspaced short palindromic repeats; EGFR, epidermal growth factor receptor; HER2, human epidermal growth factor receptor 2; nSMase2, neutral sphingomyelinase 2; TAM, tumor-associated macrophage; OXPHOS, oxidative phosphorylation

## Data Availability

No new data was generated for this review article.
